# Cotreatment with Furosemide and Hypertonic Saline Decreases Serum Neutrophil Gelatinase-associated Lipocalin (NGAL) and Serum Creatinine Concentrations in Traumatic Brain Injury: A Randomized, Single-Blind Clinical Trial

**Published:** 2018

**Authors:** Marziye Jafari, Shahram Ala, Kaveh Haddadi, Abbas Alipour, Mojtaba Mojtahedzadeh, Saeid Ehteshami, Saeid Abediankenari, Misagh Shafizad, Ebrahim Salehifar, Foroogh Khalili

**Affiliations:** a *Department of Clinical Pharmacy, Faculty of Pharmacy, Mazandaran University of Medical Sciences, Sari, Iran. *; b *Department of Neurosurgery, Emam Khomeini Hospital, Orthopedic Research Center, Mazandaran University of Medical Sciences, Sari, Iran.*; c *Department of Community Medicine, Faculty of Medicine, Mazandaran University of Medical Sciences, Sari, Iran. *; d *Department of Clinical Pharmacy, Faculty of Pharmacy, Tehran University of Medical Sciences, Tehran, Iran. *; e *Department of Neurosurgery, Emam Khomeini Hospital, Mazandaran University of Medical Sciences, Sari, Iran.*; f *Immunogenetics Research Center, Mazandaran University of Medical Sciences, Sari, Iran. *

**Keywords:** Neutrophil Gelatinase-associated Lipocalin, Serum Creatinine, Furosemide, Hypertonic saline, Traumatic brain injury

## Abstract

Acute kidney injury (AKI) occurs both after traumatic brain injury (TBI) and after hypertonic saline administration; furosemide may be useful in preventing AKI indirectly. Serum neutrophil gelatinase-associated lipocalin (sNGAL) is superior to serum creatinine (sCr) in diagnosing early AKI. We compared the administration of hypertonic saline plus furosemide (HTS+F) versus hypertonic saline (HTS), using sCr and sNGAL to investigate kidney injury in patients with TBI. This randomized, single-blind clinical trial was conducted from August 2016 to July 2017 in a neurosurgical intensive care unit, and included patients with a Glasgow Coma Score (GCS) 7-13 and brain edema. One group (n = 22) received hypertonic saline 5% (100 mL over 60 min then 20 mL/h) plus furosemide (40 mg over 60 min then 0.05 mg/kg per hour) for 72 h. The other group (n* =* 21) received only hypertonic saline 5%, in the same dose as noted above. The sCr and sNGAL concentrations, GCS, and length of stay were measured. Mean ± SD differences were -51.15 (47.07) and 9.96 (64.23) ng/mL for sNGAL and -0.12 (0.22) and -0.005 (0.2) mg/dL for sCr in HTS+F group and HTS group respectively (both *p* < 0.001). The incidence of stage one AKI according to Improving Global Outcomes (KDIGO) criteria was 4.5% in the HTS+F group and 19.0% in the HTS group (*p* = 0.16). Hypokalemia was common in both groups.

HTS+F group, compared with HTS group, was associated with lower concentrations of sCr and sNGAL. Incidence AKI (KDIGO criteria) did not have difference between groups.

## Introduction

Acute kidney injury (AKI) is one of the most important non-neurological complications of traumatic brain injury (TBI) (1, 2). Serum creatinine (sCr) has been used as a standard marker of renal function. However, because it rises 2–7 days after kidney damage has occurred, it is not useful as an early indicator of injury. Because of modest functional changes that cannot be detected by measuring, sCr could have effect on the outcome of ICU patients (3). Neutrophil gelatinase-associated lipocalin (NGAL) is the most common of biomarkers for the early detection of AKI. It is primarily secreted by neutrophils and renal proximal tubules and rises almost 2 h after injury, when damage is limited and still reversible (3-5). It is thus a useful and sensitive biomarker for the early detection of kidney injury.

Hypertonic saline is used to treat the intracranial hypertension that accompanies TBI. Electrolyte derangements, such as hypernatremia and hyperchloremia, are the most common complications associated with the use of hypertonic saline. AKI has been reported to occur during hypertonic saline infusion and hyperchloremia is recognized as a cause of AKI due to hyperchloremic metabolic acidosis (6-9).

Furosemide, a loop diuretic that increases renal blood flow and glomerular filtration rate, leads to diuresis and natriuresis, selectively inhibits sodium chloride reuptake, and reduces oxygen consumption in renal medulla. It can cause hypochloremia metabolic alkalosis. In renal tubular acidosis, furosemide also has useful impacts on the kidney. Several studies suggest that furosemide may be useful in preventing AKI indirectly and beneficial adjunct to other medical therapies, although it is controversial (10-13). 

Given that both TBI and hypertonic saline therapy can increase the risk of AKI and furosemide is thought useful adjunct to decrease renal damage, and whereas renal effect of this combination has not been evaluated, we decided to compare the effects of hypertonic saline plus furosemide (HTS+F) versus hypertonic saline (HTS) on the renal function of patients with TBI by evaluating their sCr and sNGAL concentrations. 

## Experimental

This single center, single*-*blinded, parallel-group, and randomized clinical trial was conducted in the neurosurgical ICU of Imam Khomeini Hospital, Mazandaran University of Medical Sciences, Sari, Iran, between August 2016 and July 2017. The study was approved by the research ethics committee of Mazandaran University of Medical Sciences (IR.MAZUMS.REC.94-1924, registration number IRCT01509283014N10 on July 13, 2016). The full trial protocol can be accessed at http://www.irct.ir. Written informed consent was obtained from the participants’ relatives. 

*Participants*

All patients with traumatic brain injury [Glasgow Coma Scale (GCS) score 7-13] who were transferred to the neurosurgical ICU within 24 h of sustaining a TBI and treated with hypertonic saline were recruited. The maximum interval allowed between ICU admission and start of intervention was 4 h. Eligible participants were aged 18–75 years, and had evidence of brain edema on computed tomography. Patients with brain herniation, an ejection fraction <40%, serum sodium concentration >160 meq/L or <130 meq/L, serum osmolality >350 mOsm/kg, central venous pressure (CVP) >15 mmHg, pulmonary edema, shock (mean arterial pressure (MAP) <60 mmHg), acute renal failure (increase in sCr of 0.3 mg/dL within 48 h or urine output of < 0.5 mL/kg per hour for >6 h), GFR <80 mL/min, liver failure (liver enzymes ≥ 5 times above the normal range), or pregnancy, were excluded. The patients who met the inclusion criteria were randomly assigned, using a simple randomization procedure, to 1 of 2 treatment groups. The allocation sequence was concealed from the researcher enrolling and determining allocation by using sequentially numbered opaque envelopes. The neurosurgeon, healthcare providers, and data collectors were aware of the patients’ allocations, but the outcome assessors and data analysts remained blinded to this.

**Figure 1:**
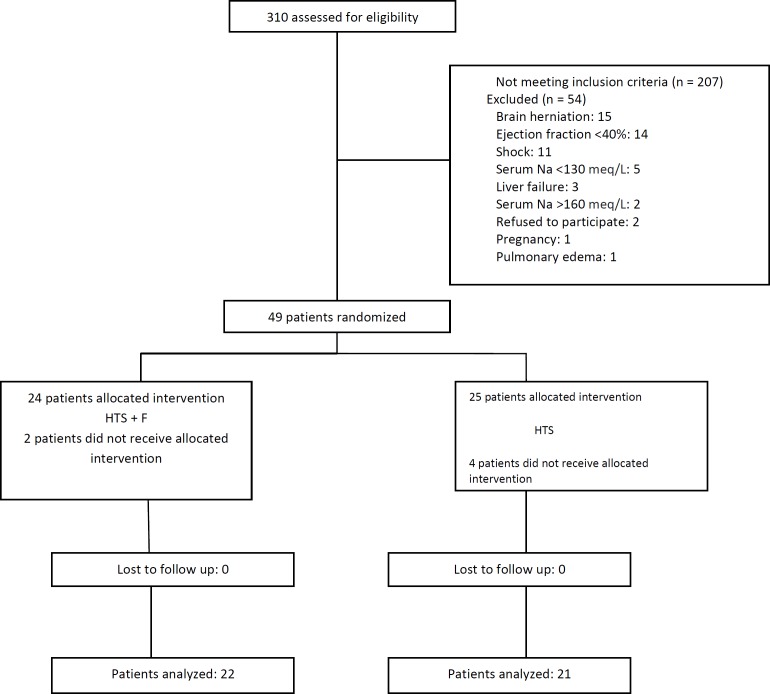
Study flow diagram

**Figure 2:**
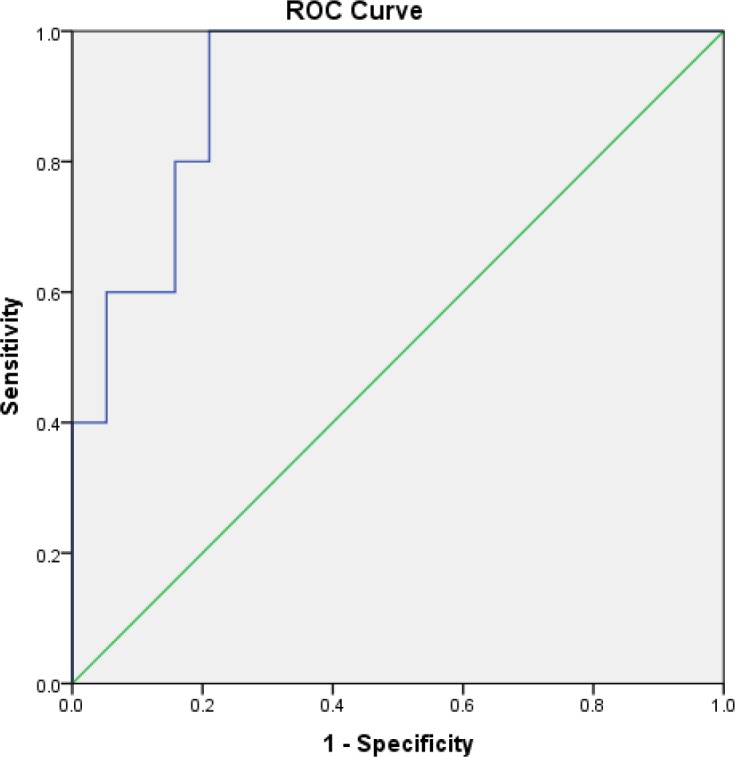
Receiver-operating characteristic (ROC) curve of Serum Neutrophil Gelatinase-associated Lipocalin (sNGAL) to detect acute kidney injury (AKI) according to KDIGO 48 h after the end of intervention. The area under the ROC curve is 0.92.

**Table 1:** Baseline demographic and clinical characteristics of patients with traumatic brain injury.

**Variable**	**Groups**		***p*** **-value** *
	HTS + Furosemide (n = 22)	HTS (n = 21)	
Age (years)	45.27 ± 16.43	44.67 ± 17.65	0.91
Weight (kg)	78.41 ± 6.97	77.86 ± 6.44	0.79
Sex F/M	4/18	2/19	0.66
Diagnosis:			
ICH/SAH	17 (77.3)	15 (71.4)	ND**
Contusion	4 (18.2)	4 (19)	
Subdural hematoma	1 (4.5)	2 (9.5)	
GCS score	9.86 ± 1.98	9.71 ± 2.3	0.8
Rotterdam CT classification:			ND**
1	4 (19)	3(15.8)	
2	6(28.6)	8(42.1)	
3	11(53.4)	8(42.1)	
APACHE II score	11.64 ± 7.15	10.82 ± 4.02	0.99
Hemoglobin (g/dL)	12.37 ± 3.60	12.58 ± 2.83	0.80
MAP (mmHg)	91.55 ± 12.05	91.90 ± 11.66	0.92
Serum creatinine (mg/dL)	0.98 ± 0.18	0.99 ± 0.16	0.87
NGAL (ng/dL)	99.99 ± 55.25	97.68 ± 54.43	0.89
Creatinine clearance (mL/min)†	102.36 ± 10.75	102.48 ± 12.25	0.97
Serum Na (meq/L)	135.80 ± 4.76	135.05 ± 5.63	0.77
Serum osmolality (mOsm/kg)	301.55±11.55	299.90 ±11.70	0.12
Urine output 24 h (L/24 h)	1.7 ± 0.42	1.5 ± 0.33	0.18

Variables are expressed as the mean ± SD or n (%).

HTS: Hypertonic saline; ICH: Intracranial hemorrhage; SAH: Subarachnoid hemorrhage; GCS: Glasgow coma score; APACHE II: Acute physiologic assessment and chronic health evaluation; NGAL: Neutrophil gelatinase-associated lipocalin.

* *p *< 0.05 considered significant,

** ND: Not determined.

† Creatinine clearance was estimated using the Cockcroft–Gault formula.

**Table 2:** Changes of variable (time **× **group effect) endpoints between groups during study.

**Variable**	**Groups**	**Time** *										**Time × group effect (p)**	
		**T0**	**T1**	**T2**	**T3**		**T4**		**T5**		**T6**		
NGAL (ng/dL)	A†	99.99 ± 55.25	48.85 ± 31.76										0.001
	B§	97.6 8 ± 54.43	107.64 ± 93.48										
SCr (mg/dL)	A	0.98 ± 0.18	1.0 ± 0.26	1.05 ± 0.19		1.0 ± 0.15	0.94 ± 0.13						<0.001
	B	0.99 ± 0.16	1.1 ± 0.20	1.25 ± 0.22		1.23 ± 0.22	1.11 ± 0.23						
pH	A	7.41 ± 0.06	7.40 ± 0.05	7.41 ± 0.05		7.41 ± 0.04	7.40 ± 0.04						0.027
	B	7.41 ± 0.08	7.40 ± 0.08	7.39 ± 0.06		7.36 ± 0.03	7.37 ± 0.06						
Na (meq/L)	A	135.50 ± 4.76	142.50 ± 7.35	144.55 ± 6.30		145.59 ± 6.76	147.36 ± 6.13	147.41 ± 5.72		147.50 ± 6.68			0.44
	B	135.05 ± 5.63	139.05 ± 6.93	143.76 ± 6.434		144.62 ± 7.345	144.33 ± 7.23	146.33 ± 6.84		147.76 ± 6.66			
Osmolality (mOsmol/L)	A	301.55 ± 11.55	301.36 ± 15.16	314.91 ± 15.53		325.91 ± 23.90	324.77 ± 12.12	312.05 ± 11.00		325.09 ± 12.41			0.45
	B	299.90 ± 11.70	294.10 ± 14.72	307.81 ± 14.71		318.19 ± 13.56	318.48 ± 12.81	308.90 ± 12.93		325.38 ± 11.36			
MAP (mmHg)	A	91.55 ± 12.05	92.41 ± 10.720	93.50 ± 11.38		93.77 ± 13.26	94.23 ± 13.86	95.50 ± 12.99		97.27 ± 13.11			0.35
	B	91.90 ± 11.66	93.33 ± 11.403	93.10 ± 10.59		96.76 ± 12.18	96.48 ± 12.83	93.43 ± 12.08		93.57 ± 11.93			

NGAL: Neutrophil gelatinase-associated lipocalin; SCr: Serum creatinine.

* Time for NGAL considered as T0: baseline, T1: day 4; time for SCr, pH considered as T0: baseline, T1: day 1, T2: day 2, T3: day 3, T4: day 4; and time for Na, Osmolality and MAP considered as T0: baseline, T1: 12 h, T2: 24 h, T3: 36 h, T4: 48 h, T5: 60 h, T6: 72 h.

† A = HTS + furosemide group.

§ B = HTS group.

**Table 3:** Adverse Events during the first 4 days of treatment for traumatic brain injury patients.

**Adverse Event** *	**HTS + furosemide (n = 22)** **n (%)**	**HTS (n = 21)** **n (%)**	***p*** **-value**
Hypernatremia (>157 meq/L)	0	1 (4.7)	0.33
Hypokalemia (<3.5 mEq/L)	10 (45)	6 (26)	0.42
Coagulopathy	2 (9.1)	2 (9.5)	0.96
Rash	1 (4.5)	0	0.32
Acute hypotension	1 (4.5)	0	0.32
Thrombophlebitis	2 (9.1)	1 (4.7)	0.59
Thrombocytopenia	2 (9.1)	3 (14.2)	0.59
Swelling at the injection site	2 (9.1)	1 (4.7)	0.58

* Values are numbers (percentages). The proportions of patients with adverse events were compared between groups by using chi square.

*Intervention*

The HTS+F group received hypertonic saline 5% (loading dose of 100 mL over 60 min followed by an infusion of 20 mL/h for 72 h) and furosemide (20 mg/2 mL: loading dose 40 mg over 60 min followed by an infusion of 0.05 mg/kg per hour for 72 h). The HTS group received hypertonic saline 5% (loading dose of 100 mL over 60 min followed by an infusion of 20 mL/h for 72 h). All patients were treated according to the hospital’s TBI guideline (14). Hemodynamic parameters were constantly monitored. Volume resuscitation was achieved with 0.9% normal saline to maintain central venous pressure (CVP) at 8–12 mmHg. After adequate fluid resuscitation, the MAP was kept above 90 mmHg. In both groups, the hypertonic saline infusion was stopped when the serum sodium and serum osmolality reached more than 157 meq/L and 320 mOsm/kg respectively.

*Measurements*

Patient demographics, survival, mortality, and length of ICU and hospital stay were recorded. The GCS and Acute Physiologic Assessment and Chronic Health Evaluation (APACHE II) scores, MAP, serum osmolality, as well as blood pH were recorded at baseline and daily up to day 4. Serum sodium and potassium concentrations were measured 12-hourly. CVP was measured 6 hourly.

AKI was defined using the Kidney Disease: Improving Global Outcomes (KDIGO) criteria and was assessed up to 48 h after discontinuation of treatment. The stages of AKI, based on the KDIGO classification, were as follows: Stage 1: serum creatinine ≥0.3 mg/dL (in 48 h) or 1.5–1.9 times the baseline value (in 7 days); Stage 2: serum creatinine 2–2.9 times the baseline value; and Stage 3: serum creatinine ≥3 times the baseline value, or ≥4 mg/dL. Several sNGAL cut-off points can be used to determine AKI; we used a cut-off value of >150 ng/mL to predict AKI in patients with TBI (3, 15 and 16). Adverse events were documented by observation or as reported by the health care provider during the study period. 

For biomarker measurement, a 5-mL blood sample was collected via the patients’ central venous catheters at baseline and on day 4. Each blood sample was stored at room temperature for approximately 30 min and was then centrifuged at 3,500 rpm for approximately 20 min. The serum samples were isolated and stored at -80 °C until analysis. SNGAL levels were determined via enzyme-linked immune-absorbance assay (ELISA) kits (Bioassay Technology Laboratory, Shanghai Korain Biotech), according to the manufacturer’s instructions; the assay is a sandwich ELISA performed in microwells coated with a monoclonal antibody against mouse NGAL. Bound NGAL is detected with another monoclonal antibody labeled with biotin and the assay is developed with horseradish peroxidase (HRP)-conjugated streptavidin and a color-forming substrate.

Laboratory investigators were blinded to sample sources and clinical information until the end of the study.

*Outcome variables*

The primary outcome variables were the degree of increase in sNGAL and sCr concentrations from baseline to peak concentrations, and the incidence of AKI according to both KDIGO criteria and sNGAL concentration (>150 ng/mL) within 4 days of initiating the interventions. Secondary outcomes included changes in blood pH, MAP, length of ICU and hospital stay, and survival duration. 

*Sample size calculation*

Based on data from an observational pilot study conducted in our ICU that indicated a mean difference in sNGAL between the HS and HS+F groups of 30 ng/mL (SD, 33 ng/mL) on day 4, we anticipated that 19 patients would be required in each arm (80% power and a significance level of 0.05). Allowing for 20% attrition, we aimed to recruit 46 patients.

*Statistical analysis*

Descriptive baseline characteristics for each group were compared and tabulated as the mean ± SD for normally distributed continuous variables or as frequency and proportion for categorical variables. All analyses comparing the efficacy of the primary outcomes were conducted under the intention-to-treat principle. Using a general linear model score, those parameters were compared between the 2 groups using the repeated measure ANOVA test. The assumption of compound symmetry was examined using Mauchley’s sphericity test. The time groups’ cross-products (interaction terms) were considered when analyzing the differences between the groups in their responses over time; the baseline values (age, weight, GCS, and primary diagnosis) were the covariates in this model. Generalized Estimating Equation (GEE) model was used to estimate the differences in the outcome values at each time point between the two groups as well as the trend of time after treatment. Correlations between quantitative variables were evaluated using Spearman’s test. Survival analysis (Kaplan-Meier method) and log-rank tests were used to estimate the mean survival time ± SEM, stratified by group. For all analyses, the statistical software IBM SPSS Statistics for Windows, Version 21.0 (Armonk, NY: IBM Corp.), was used. All statistical tests were 2-tailed, and a significance level of 5% (alpha = 0.05) was used.

## Results

Of the 310 patients with TBI referred to our hospital, 207 did not meet the inclusion criteria. The 43 remaining patients were randomly allocated into the 2 groups 22 to the HTS+F group and 21 to the HTS group (Figure 1) and their data were analyzed. The study population was 37 (86%) male and mean ± SD age of 44.98 (16.84) years. Other patients’ basic demographic and clinical characteristics are shown in Table 1; (all *p* > 0.05).

*Primary outcome*

Level of sCr and sNGAL were evaluated at various time points and compared between the groups (Table 2). Although the level of sNGAL decreased in the HTS+F group on day 4, it increased in the HTS group on day 4. Mean ± SD differences in HTS+F group and HTS group were -51.15(47.07) and 9.96 (64.23) ng/mL respectively (*p* < 0.001). After correcting for possible confounding variables (age, sex, and osmolality) in GEE model , revealed that sNGAL concentration was 33.19 ng/mL higher the HTS group than the HTS+F group (CI 95%: 3.33-63.04 ; *p *= 0.03).

SCr concentration increased in the HTS group but almost remained constant in the HTS+F group. Mean ± SD difference (T_1_-T_4_) in HTS+F group and HTS group were -0.12 (0.22) and -0.005 (0.2) mg/dL respectively (*p* < 0.001). After correcting for possible confounding variables (age, sex, and osmolality) in GEE model, it was revealed that the HTS+F group patients have 0.098 mg/dL sCr higher than the HTS group patients (CI 95%: 0.001-0.187; *p *= 0.03).

27.3% (6 patients) in the HTS+F group and 23.8% (5 patients) in the HTS group had sNGAL concentrations >150 ng/mL at baseline. Incidence of AKI according to sNGAL concentrations >150 ng/mL 4.55% (one patient) in the HTS+F group and 23.81% (5 patients) in the HTS group on day 4 respectively (*p *= 0.04, power = 77%).

 According to KDIGO criteria , stage 1 AKI was developed 4.5% (one patient) and 19% (4 patients) in the HTS+F group and in the HTS group at follow up time respectively (*p *= 0.16, power = 32%).

From the 6 patients in the HTS+F group and 5 patients in the HTS with sNGAL concentration >150 ng/mL at baseline, 16% (one patient) and 80% (4 patients) developed AKI according to KDIGO criteria respectively. All of the patients who progressed to AKI had sNGAL concentration >150 ng/mL at baseline. Accuracy of sNGAL level at for AKI diagnosis was evaluated in Roc curve (Figure 2).

As shown in Figure 2 sNGAL concentration with cut of point 122.45 was suitable (sensitivity = 100%, specificity = 79%, AUC in Roc curve = 0.92, *p *= 0.003).

*Secondary outcome*

Although blood pH was in the normal range during the study period in both groups, this parameter decreased significantly in the HTS group (*p* = 0.03) (Table 2). The maximum decline in pH was observed on day 3 in the HTS group. The MAP decreased in both groups, with no significant difference between the groups (*p* = 0.93) (Table 2). There were not significant differences in terms of length of ICU stay (5.5 vs. 6 days, respectively, *p = *0.4) and length of hospital stay (7.5 *vs.* 8 days, respectively, *p* = 0.14). The hospital mortality was 3 patients and 4 patients in HTS+F group and HTS groups respectively .The mean survival durations of patients in the HTS+F and HTS groups were 27.1 (SEM, 1.5) and 26.3 (SEM, 1.6) days, respectively (*p *= 0.67). 

Mean serum sodium and osmolality were virtual identical in both groups and both were increased, and did not have difference between groups (Table 2).

Treatment was discontinued in 1 patient in the HTS group because of hypernatremia; all adverse events are shown in Table 3. 

*AKI and no AKI patients (KDIGO criteria and* NGAL > 150 ng/mL*)*

At baseline, the mean sCr concentrations were 0.99 ± 0.15 mg/dL and 0.90 ± 0.25 mg/dL for patients without AKI and with AKI, respectively (*p* = 0.89). 

There were significant differences between patients with and without AKI (KDIGO criteria) in terms of length of ICU stay (5.8 vs. 8.6 days, respectively, *p = *0.009*) *and length of hospital stay (7.8 *vs.* 10.4 days, respectively, *p *= 0.003), but mortality rates were not different (*p *= 0.34). Patients with AKI (KDIGO criteria) had greater proportion received clindamycin (80% *vs.* 29%, respectively, *p* = 0.02).

There were significant differences between patients with baseline sNGAL concentrations >150 ng/mL and <150 ng/mL in terms length of hospital stay (7.7 *vs.* 9.5 days, respectively, *p* = 0.03), but mortality rates were not different (*p *= 0.43).

## Discussion

To our knowledge, this is the first study to compare the combination of bolus and continuous infusion of hypertonic saline plus furosemide versus hypertonic saline alone in patient with TBI, and to compare sNGAL and sCr as marker for diagnostic and monitoring purposes. This study’s most notable finding was that sNGAL and sCr concentrations decreased in the HTS+F group, and also blood pH level decreased significantly in the HTS group. Other important finding is that the incidence of AKI (KDIGO criteria) in the HTS group was 4-fold higher than in the HTS+F group; although there was not a statistically significant difference, incidence of AKI according to sNGAL concentrations >150 ng/mL in the HTS group is statistically higher than in the HTS+F group.

Over the past decade sNGAL has become a marker for early detection of AKI, as its concentration increases within a few hours post-insult. Serum or urine levels of NGAL correlate with the severity of AKI. Patients with elevated sNGAL concentrations, even those with normal sCr concentrations, seem to have an increased risk of mortality or of requiring renal replacement therapy (3-5). McIlroy *et al.* suggested that chronic kidney injury reduced the accuracy of sNGAL in predicting AKI, with sNGAL being best identifying AKI in patients with a baseline glomerular filtration rate (GFR) of 90–120 mL/min (17). As our patients had normal renal function (GFR: 80–120 mL/min) at baseline, they were almost homogeneous and good choice for evaluating sNGAL. There is no consensus agreement regarding NGAL cut-off point to define AKI, but several sNGAL cut-off points are used to determine AKI. Some studies suggest that sNGAL concentrations <100 ng/mL and >150 ng/mL are useful to exclude and diagnose AKI, respectively (3, 16). We chose a cut-off of >150 ng/mL as a potential risk factor for kidney injury. In our study, all patients who developed stage one AKI (KDIGO criteria) had baseline sNGAL concentrations of >150 ng/mL, and sNGAL increased earlier than sCr did. 

Although the sNGAL concentration displayed greater variation than did the sCr concentration at baseline, it indicates that TBI can lead to renal damage or systemic generation of sNGAL as a stress reaction, regardless of the type of treatment administered. This finding is in keeping with those of previous studies (1, 5). It can explained by brain–kidney cross-talk (18) (brain damage progresses to the syndrome of multiple organ dysfunction, a syndrome that is likely mediated by dysregulated inflammatory mechanisms) and rise of sNGAL after inflammatory condition (4, 19). 

Although the level of sNGAL was >150 ng/mL in some patients at baseline, after our intervention, the sNGAL concentration significantly decreased in the HTS+F group but increased in the HTS group. It seems that this was related not only to TBI but also to other factors.

Changes in sCr concentrations are more reliable than sNGAL, because there is no consensus agreement for NGAL cut-off point, sCr is not affected by environment stress and systemic generation. In our study, sCr concentrations increased in the HTS group and it was significantly different on day 2 and 3 compared to HTS+F group.

The use of mannitol and hypertonic saline, coagulopathy, sepsis, rhabdomyolysis, blood loss, contrast agents and antibiotics was associated with rise sCr and risk of kidney injury 

(2, 20-22).

Many studies have shown hypertonic saline to be therapeutically beneficial in TBI, but its effects on the kidney remain controversial (6, 8, 9 and 23). Hypertonic saline (especially continuous infusion), and chloride-containing solutions can cause systemic complications such as hyperchloremia, metabolic acidosis, and AKI (9, 18). Kelly *et al*. investigated the effect of hypertonic saline on patients with severe TBI (19). AKI occurred in 12.1% of patients in the continuous infusion group but did not occur in the bolus group. In another study, patients with burns were treated with hypertonic saline or Ringer lactate solutions; renal failure and mortality were 4 and 2 times higher, respectively, in the group treated with hypertonic saline (24).

The mechanisms by which hypertonic saline induces kidney injury are unclear, but several hypotheses have been proposed. First, chloride-containing solutions, via mesangial contraction, increase chlorine absorption and induce renal vasoconstriction (18, 24 and 25). Chloride also induces thromboxane production; thromboxane causes renal vasoconstriction and reduced renal perfusion delays the onset of diuresis (9). Second, it may increase the risk of hypernatremia and hyperchloremia, causing an osmotic gradient resulting in fluid shifts from the endothelial cell to the interstitial space (6, 24 and 26). This hypothesis is acceptable as low chloride solutions reduce the AKI risk in ICU patients regardless of the underlying disease (9).

To date, none have evaluated this combination in humans as a form clinical trial. A few studies have combined furosemide with hypertonic saline or mannitol in rats and metabolic acidosis was reported in the hypertonic saline group versus furosemide with hypertonic saline (27). In our study, blood pH decreased in both groups, but was significantly lower in the HTS than in the HTS+F group. This hypothesis can be proven that hypertonic saline-induced acidosis may lead to an increase in sNGAL and sCr concentrations. Patients with high baseline levels of sNGAL following TBI were more sensitive to develop hypertonic saline-induced metabolic acidosis, but concomitant administration of furosemide and hypertonic saline had positive effects on the kidney and reduced the concentration of sCr and sNGAL; however, no significantly difference in outcome was found between the 2 groups. 

Several studies have demonstrated the effects of furosemide on the kidney (10, 12). One meta-analysis found that the combination of furosemide and hydration reduced contrast-induced AKI in patients undergoing angiography (12); another found that furosemide may be more effective in mild AKI than in severe AKI (10). In terms of evidence-based pharmacology, loop diuretics act on the Na^+^/K^+^/2Cl^−^ cotransporter in loop of Henle, thereby reducing oxygen demand in medulla. Furosemide also improves renal cytochrome oxygenation, and enhances the rate of elimination of toxins and casts from the tubules. Furosemide stimulates prostaglandin production and reduces ischemia via renal vasodilation. Finally, in contrast to hypertonic saline-induced hyperchloremic metabolic acidosis, furosemide causes a metabolic alkalosis (10, 11, 28 and 29). Therefore, furosemide may reduce hypertonic saline induced kidney injury via inducing a metabolic alkalosis and via other mechanisms mentioned. 

We also identified clindamycin as a risk factor; few studies have reported an association between clindamycin and AKI (30). Hypokalemia was the most common adverse event in our study*.* Disruption of sodium and potassium exchange in the distal tubule, caused by both hypertonic saline and furosemide, leads to hypokalemia (28, 31). 

Our study has some limitations. First, it was restricted to one center, the data were open-label, and the clinicians and data collectors were not blinded. Second, sNGAL is also a stress marker; its concentration increases after infectious, inflammatory, and ischemic insults. Third, some researchers have shown that urinary NGAL may be a better marker of intrinsic AKI than sNGAL, but we were not able to measure urinary NGAL concentrations. Fourth, as mentioned before**, **there is no consensus agreement regarding NGAL cut-off point to define AKI. Changes in its serum concentrations (especially urine concentrations) are more reliable. A cut-off value of 150 ng/mL for sNGAL concentration seems arbitrary albeit reasonable, but our findings may have been different had we chosen a different cut-off value. Fifth, the incidence of AKI may have been underestimated; because of we excluded patients with renal impairment to improve the reliability of sNGAL in evaluating AKI. Lastly, the study does not reflect the effect of hypertonic saline plus furosemide on preventing AKI—this needs further exploration and larger sample size.

In conclusion, TBI can lead to an early increase in sNGAL concentration. The use of hypertonic saline plus furosemide, compared with hypertonic saline alone, was associated with lower sNGAL and sCr concentrations. There was no significant difference between groups regarding AKI incidence and using hypertonic saline plus furosemide in preventing AKI requires further research.
